# Measuring Human Memory B Cells in Autoimmunity Using Enzyme-Linked ImmunoSpot

**DOI:** 10.3390/biom15050643

**Published:** 2025-04-30

**Authors:** Georgia Stylianou, Greg A. Kirchenbaum, Paul V. Lehmann, Simon Pearce, Stephen Todryk

**Affiliations:** 1 Faculty of Health & Life Sciences, Northumbria University, Newcastle upon Tyne NE1 8ST, UK; georgia3.stylianou@northumbria.ac.uk; 2 Translational and Clinical Research Institute, Newcastle University Centre for Life, Central Parkway, Newcastle upon Tyne NE1 3BZ, UK; 3 Cellular Technology Ltd., 20521 Chagrin Blvd., Shaker Heights, OH 44122, USA; greg.kirchenbaum@immunospot.com (G.A.K.); paul.lehmann@immunospot.com (P.V.L.)

**Keywords:** B cell ELISPOT, anti-self antibodies, antibody-secreting cells, peripheral blood mononuclear cells (PBMCs)

## Abstract

The measurement of serum antibodies that specifically recognize self-antigens is a critical diagnostic in autoimmunity. A limitation of such an approach is sensitivity to detect the antibody, particularly when abundant self-antigens in the body may bind and sequester circulating specific antibodies. The presence of specific memory B cells (B_mem_) may provide a more sensitive and robust indicator of an autoimmune response, as is suggested for certain anti-viral responses. B cell enzyme-linked ImmunoSpot (ELISPOT) is capable of detecting antigen-specific B_mem_ cells in blood at the single cell level, following stimulation of peripheral blood mononuclear cells (PBMCs) to expand and differentiate the B_mem_ cells into functional antibody-secreting cells (ASCs). While this assay has been widely utilized in infectious diseases and vaccination, detection is more difficult for autoantigens due to self-tolerance and specific tissue compartmentalization of immune responses, making autoantigen-specific B cells rare in the circulation. The cycles of re-activation of B_mem_ cells to become ASCs, that may reflect disease flare-ups in autoimmunity, are not well defined. For several autoimmune diseases (ADs), the targeting of B cells via depleting monoclonal antibodies has proven to be an effective treatment, where B_mem_ cells are likely being targeted. The measurement of autoantigen-reactive B_mem_ cells may aid in diagnosis and staging of clinical severity, or be a metric for efficacious treatments, thus providing an additional informative biomarker of ADs. How B cell ELISPOT has been utilized to characterize B_mem_ cells in human ADs is described here, including the advantages and disadvantages of the assay.

## 1. Introduction

The measurement of circulating antigen-specific antibodies in the blood is a mainstay approach for determining beneficial immunity against pathogens in the context of infection or vaccination. This is also the case for anti-self antibodies in most autoimmune diseases (ADs), but where such antibodies are either directly detrimental, inducing an attack on self-tissues, or are a surrogate marker of autoimmunity where T cells have the direct pathogenic effect. Interestingly, B cells are often the target for therapy through their depletion via anti-CD20 monoclonal antibodies in a wide range of ADs, even where involvement of autoreactive B cells was not considered to be the main pathogenic effector mechanism. However, owning to the therapeutic success of B cell-depleting therapies in the context of several autoimmune diseases (ADs), it has become increasingly clear that B cells are critically involved in disease activity [1,2,3,4,5]. Furthermore, removal of autoreactive antibodies via plasmapheresis is also a treatment for some ADs. Anti-self antibody responses cover a range of specificities and magnitudes depending on the AD; and in the clinical setting, they are typically measured using one of several immunoassays ranging from immunohistochemistry and radioimmunoassay to ELISA and high-throughput systems using chemiluminescence such as the Roche COBAS.

Loss of immune tolerance to self-antigens in ADs requires that multiple immune checkpoints fail, and continue to do so, for the active production of autoantibodies [6]. Despite the ubiquitous nature and abundance of some self-antigens, potentiating autoimmune reactions, specific autoantibodies may become sequestered from circulation and accumulate in tissues, and this is an underappreciated challenge for their detection using traditional serologic methodologies. Furthermore, ASCs against autoantigens may predominantly reside in the particular tissues expressing the autoantigen. In some instances, anti-self antibodies may possess a reduced affinity, and this further complicates their detection, especially when present at low titers in circulation. Low-affinity BCR on B cell clones enables escape from immune tolerance mechanisms. In contrast, autoantibodies with high affinity, such as found in Graves’ Disease, are potent at causing disease at very low concentrations. While the pathologic consequences of autoantibodies—secretory products of self-reactive plasma cells—have been studied extensively, the role of autoreactive memory B cells (B_mem_) that represent an alternative lineage than terminally differentiated plasma cells [7] remains to be elucidated despite the success of B cell depletion therapies [8,9].

**Figure 1:**
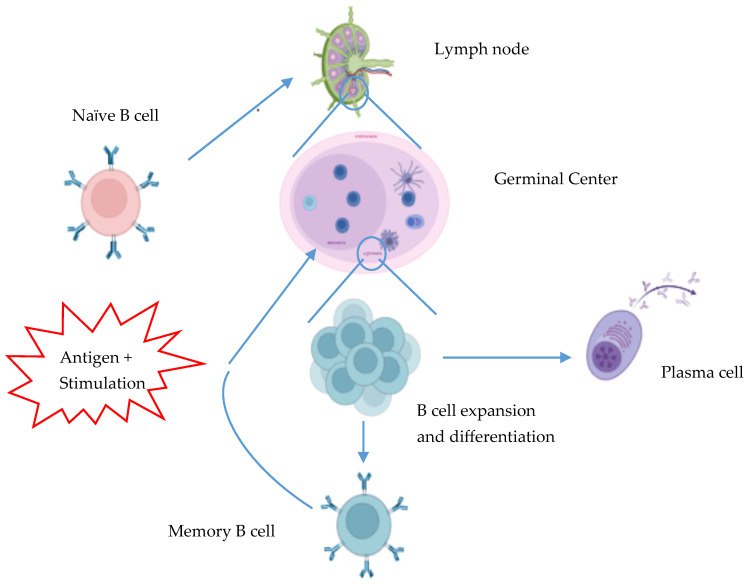
B cell priming and differentiation. Upon encounter with pathogen or vaccine antigens, naïve B cells will enter the germinal center and undergo clonal expansion and class-switch recombination. Some of those B cells become short-lived IgM-secreting cells and serve as the first line of defense. Whereas, the B cells that will remain in the germinal center will undergo somatic hypermutation and additional class-switch recombination to increase their affinity for the antigen. Then, with additional clonal expansion and selection, these specific B cells will ultimately exit the geminal center as plasma cells or as B_mem_ cells that constitute immunological memory.

Ideally, a single exposure to an infectious pathogen, or an antigen via vaccination, will result in lifelong immunity through the generation of immunological memory, comprising clonally expanded T cells capable of killing infected cells and B cells (Figure 1) that secrete or have the capacity to secrete specific antibodies. The antibody-secreting cells (ASCs) can be further characterized as short-lived or long-lived plasma cells, while the long-lived memory B_mem_ cells are quiescent and retain the capacity to secrete antigen-specific antibodies upon antigen re-encounter. Thus, a subsequent exposure to the same pathogen/antigen will trigger B_mem_ cells to rapidly proliferate and differentiate into ASCs regardless of whether they re-enter into a germinal center. Moreover, germinal center B cells with higher affinity for the antigen will preferentially be selected for differentiation into plasma cells, while B subclones endowed with BCR of reduced affinity are more likely to enter into the B_mem_ compartment. Hence, this subsequent exposure to the same antigen not only generates an enhanced immune response comprising an increased production of antibodies that are even more effective against the pathogen, enabling the overcoming of a larger challenge of this pathogen and in less time, but also the accommodation of antigen variants required for combatting mutant viruses. In the case of autoimmunity, such a process likely maintains or increases pathogenic autoantibody levels even further but also causes diversification of the autoimmune memory B cell repertoire. This is shown by the expansion B cell clonal lineages, along with the acquisition of somatic hypermutations within the *IgH/IgL* genes, in many ADs [1,10].

The process of generating antigen-specific B cells with unique antigen specificities initially occurs primarily in the bone marrow where *V(D)J* recombination of the B cell receptor (BCR) genes takes place, in association with clonal deletion of self-reactive B cells (central tolerance) that failed to generate an innocuous BCR through receptor editing [11]. Despite the remaining autoreactive B cells undergoing anergy outside the bone marrow (peripheral tolerance), autoreactive B cells remain and produce antibodies that may precipitate autoimmune pathology. Exclusion of B cells from follicles of secondary lymph nodes and germinal centers, and lack of cognate T cell help, may further aid tolerance.

During the primary immune response, naïve B cells with a specific BCR are activated to class-switch, undergo affinity maturation, and differentiate and proliferate into ASCs and memory cells (Figure 1). This process most efficiently occurs in the germinal centers of secondary lymphoid tissues with support from helper T cells that provide CD40 ligation and secrete activatory cytokines including IL-21 [12]. The primary immune response consists largely of short-lived plasmablasts; however, longer-lived plasmablasts (plasma cells) also arise and remain in the circulation of the host for a few weeks, depending on the magnitude of the immune response. Plasma cells mainly home to and reside in niches within lymphoid tissues such as the bone marrow, where they secrete specific antibodies into the circulation (Figure 1). Simultaneously, during a primary immune response, B_mem_ cells with the same specificity will be generated that have the capacity to become antibody-secreting cells (ASCs) and to increase their affinity for antigen upon future antigen re-encounter. This generation of ASCs and B_mem_ cells appears to be a continuous and unregulated process in ADs, and for which, the initiation events are largely unknown. Some theories suggest viral infections initiate immune responses in ADs, while others demonstrate genetic polymorphisms that disrupt key immune regulatory molecules. ADs clearly have a multifactorial etiology.

Since it proved to be suitable for measuring immunity to infectious diseases and vaccination [13,14,15,16,17], B cell ELISPOT can also potentially detect the magnitude and dynamics of key immune responses in ADs, which may be informative for clinical decisions. This review aims to describe the efforts at utilizing B cell ELISPOT as a biomarker in a range of autoimmune diseases.

## 2. B Cell ELISPOT in ADs

**Figure 2:**
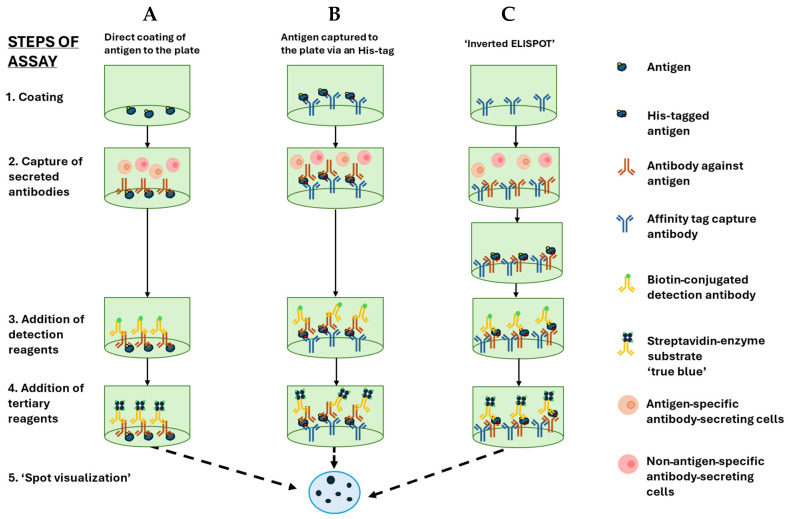
Schematic representation of the B cell ELISPOT assay. The B cell ELISPOT assay is composed of five main steps: 1. Coating of antigen, affinity capture of antigen, or total Ig capture; 2. addition of ASCs and capture of secreted antibodies; 3. addition of detection reagents (detection antibody followed by conjugate); 4. addition of substrate; and 5. spot visualization, imaging, and analysis. Three main forms of the B cell ELISPOT assay are distinguished by their application of antigen. The first column represents the steps involved in a direct coating of the antigen onto the PVDF membrane. The second column involves affinity coating of the antigen via an anti-His antibody rather than directly onto the membrane. The last column represents the steps involved in an inverted assay that involves capturing all secreted antibodies and then probing with the specific biotinylated antigen followed by SA-ALP and substrate.

The B cell ELISPOT assay was initially described in 1983 in parallel by Sedgwick [18] and Czerkinsky [7] and has since been developed into an effective tool to quantify ASCs [16]. The B cell ELISPOT assay was first used in the context of autoimmunity to detect B cells secreting rheumatoid factor in Rheumatoid Arthritis (RA) patients [8]. Soon after, it was used to detect B cells secreting self-antigen-specific antibodies not only in RA but also in Sjögren’s Syndrome (SS), Myasthenia Gravis (MG), Ulcerative Colitis (UC), and Systemic Lupus Erythematosus (SLE) [9,10,11,12,13,14]. ELISPOT was also utilized to enumerate T cell responses through cytokine capture, and the introduction of the polyvinylidene fluoride (PVDF) membrane for improving coating density of the anti-cytokine capture antibodies within specialist 96-well microtiter ELISPOT plates was a major step in developing the ELISPOT assay that led to clear reproducible spots representing responding cells [15]. Simultaneously, instruments and software for analysis have been developed for counting spots with high accuracy, reducing human error and providing great reproducibility [15]. Similarly, the B cell ELISPOT has been refined, with the standard methods for B cell ELISPOT shown in Figure 2. The principal method involves coating the PVDF membrane of wells directly with a defined antigen. This may be a mixture of proteins (such as a tissue lysate), purified proteins, or recombinant proteins [19,20,21]. Frequently, however, direct coating with the antigen of interest cannot be accomplished by direct adsorption; in such cases, affinity coating permits detecting the antigen-specific B cells [20]. Recombinant proteins can readily be engineered to include affinity tags, such as 6-Histidines, which enable the protein to be affinity coated more effectively compared to direct adsorption to the PVDF membrane [16]. Moreover, another benefit of affinity coating is that it likely allows for the coated antigens to better retain their native structure. Such affinity-tagged recombinant proteins can also be used as detection probes in an “inverted” B cell ELISPOT assay, in which secretory footprints from individual ASCs are captured irrespective of their specificity using pan-Ig capture reagents. High content analysis of spot morphologies can also shed insights into the affinity spectrum of the antigen-specific repertoire; however, for this application of the assay, the FluoroSpot approach is most effective [17].

Distinct from an enzyme-linked immunoassay (ELISA), which is restricted to measuring the bulk serum antibodies produced in vivo by antibody-secreting plasma cells, ELISPOT can not only measure ASCs directly ex vivo but also B_mem_ cells within PBMC. Since B_mem_ cells are quiescent and do not actively secrete their BCR as antibody, an in vitro polyclonal stimulation is required to trigger their terminal differentiation [22]. Numerous studies have been successful in utilizing the B cell ELISPOT assay in infectious diseases such as SARS-CoV-2, Epstein–Barr Virus (EBV), Cytomegalovirus (CMV), and seasonal influenza to detect antigen-specific responses [13,14,15,16,17]. In the area of ADs, there seem to be far fewer research studies utilizing B cell ELISPOT to track disease progression and severity. This is despite several studies showing B cell ELISPOT as a detection assay for ASCs in Multiple Sclerosis (MS), Hashimoto’s Thyroiditis (HT), Type 1 Diabetes (T1D), and Rheumatoid Arthritis (RA) [18,19,20,21]. Most studies focus on the circulating B_mem_ cells found in the PBMC of the donors that, upon stimulation with an activator of B cells (such as R848 [TLR7,8 agonist] and IL-2), differentiate into measurable ASCs [16,23]. Crucial to utilizing the B cell ELISPOT assay is the choice of antigen to use when measuring ASCs. In order for a ‘secretory footprint’ to appear, a specific antigen/protein must be effectively adsorbed directly onto the PVDF membrane or via a capture antibody in the case of affinity coating (Figure 2). Briefly, once the antigen is captured, and after washes and blocking of the plate, the antibodies secreted by the B_mem_-derived ASCs (stimulated PBMCs) or plasma cells (unstimulated PBMCs), added to the plate, will bind to the captured antigen just in the close vicinity of the ASCs. The cells are then washed off, and a biotin-conjugated detection antibody is added, followed by a streptavidin–enzyme conjugate, again interspersed with washing. This then allows substrate deposition to be used to visualize the secretory footprint, a spot, produced by the individual ASCs. Titration of cells is required if the ASCs are in high abundance in order to obtain discrete, countable spots. The Handbook of ELISPOT provides extensive detail and protocols of ImmunoSpot assays (collective name for ELISPOT and FluoroSpot) for assessing the antigen-specific B cells [6].

ELISPOT has proven to be more sensitive than serum antibodies in identifying anti-viral immunological memory for certain individuals [14]. Given the importance of antibodies, and thus ASCs, in driving the pathophysiology of several ADs, B cell ELISPOT may provide an accurate representation of disease state and progression and response to treatments particularly that target B cells. A major component of the ELISPOT assay is identifying the ideal antigen to use when measuring ASCs in ADs. Table 1 describes the major autoimmune diseases, in which anti-self antibodies are considered important as effectors or markers of disease, and the antigens against which antibodies are measured. Therapy for these ADs targeting B cells, particularly B cell-depleting therapy, has become an effective treatment option in the last decade. Rituximab, Ocrelizumab, and Ofatumumab are anti-CD20 monoclonal antibodies that deplete B cells [1]. Epratuzumab targets CD22 on B cells for depletion, while Belimumab neutralizes soluble B cell activating factor (BAFF) in circulation [8,24,25]. However, it remains unclear precisely how CD20^+^ B cell depletion treatment improves ADs. Since ASCs that produce antibodies in vivo (plasma cells) typically do not express CD20, it is suggested that anti-CD20 mAb treatment depletes B_mem_ cells that are the source of autoreactive ASCs. Often, antibodies are known to be the prime effector molecules involved in pathogenesis (Graves’ Disease and Hashimoto’s Thyroiditis) and where B cell-depleting therapies are logical. Traditionally in MS, antibodies were not considered the pathogenic effector molecules (although later, EAE data prove differently [19]), and also the fact that the disease is treatable by B cell depletion therapy points towards a pathological mechanism that involves B cells [1,26]. As well as autoantibody production, B cells may be contributing to pathogenic anti-self T cell responses by efficiently presenting the self-antigens to the T cells or by contributing to the formation of conventional and ectopic lymphoid tissues within target tissues. Thus, antibodies and ASCs may be useful surrogate markers of disease. Overall, improved monitoring of B cell responses may help inform disease trajectory and indicate the cessation of depletion therapies that have side effects such as immune suppression and resulting increased infections.

## 3. Examples of Diseases in Which Autoantibodies Are Effectors of Disease Pathogenesis

### 3.1. Rheumatoid Arthritis (RA)

Rheumatoid Arthritis (RA) is an AD in which patients initially present with inflammation of the synovium, damage to the cartilage, and erosion of the bone that eventually leads to joint destruction causing permanent disability. Many factors drive the pathogenesis of the disease including autoreactive T and B cells, M1 macrophages, neutrophils, and inflammatory cytokines [27]. Similarly to secondary lymph nodes, the synovial tissue that lines joints also contains T and B cell differentiation sites known as synovial ectopic lymphoid structures [56]. The are several types of antibodies found in RA patients including rheumatoid factor (anti-IgGFc), anti-perinuclear factor (APF), anti-keratin (AKA), anti-collagen, anti-nuclear proteins, anti-MCV, and anti-citrullinated protein antibodies (ACPAs [including Vimentin]) [56,57]. Initially, the disease was considered to be a B cell-driven AD due to high amounts of IgM autoantibodies known as rheumatoid factor (RF). RF is an antibody targeting the Fc region of IgG that leads to the formation of immune complexes with the IgG antibodies in the joint and is usually associated with poor prognosis [58]. However, recent studies have shown RF is not found in all confirmed cases of RA patients, while spontaneous production of ACPAs can be detected in healthy individuals [59].

In most cases, RA is driven by ACPAs produced by B cells. B cells producing ACPAs recruit neutrophils through the secretion of Interleukin-8, whereas the antibodies themselves promote an immune response against proteins found in the synovial tissue (e.g., citrullinated filaggrin, fibrin, vimentin, enolase, and collagen) [27,59]. Cross-reactivity between post-translationally modified proteins and foreign antigens from microorganisms can increase the population of autoreactive B cells in RA patients [27]. Simultaneously, the inflamed joint provides the ideal environment for the production of memory B cells (CD20^+^, CD38^−^) that differentiate into ASCs. ASCs from the inflamed joint area will eventually migrate to the bone marrow and become anti-CCP plasma cells [57].

Undoubtedly, autoreactive B cells and autoantibodies are effectors driving the pathogenesis and disease progression in the context of RA. The success of B cell depletion therapies such as Rituximab in RA has further supported autoantibodies to be effectors in RA [27]. ACPA and RF autoantibodies are utilized as markers for disease diagnosis and disease progression, whereas erythrocyte sedimentation rate (ESR) and C-reactive protein (CRP) are used for inflammatory disease activity [60]. A recent study by Reijm et al. investigated the post-translational modification of self-antigens citrulline, homocitrulline, and acetyl lysine and their recognition by B cells. The study was successful in identifying several B_mem_ cell populations while also noting large populations of ASCs against the same antigens [61]. A study by Kerkman et al. utilized B cell ELISPOT to investigate the longevity and resilience of unstimulated autoreactive ACPA-IgG-secreting PCs and showed them to remain stable for up to 6 months in vitro [62].

### 3.2. Systemic Lupus Erythematosus (SLE)

SLE is a multiorgan chronic autoimmune disease characterized by dysregulated adaptive and innate immune responses [63]. Patients with SLE suffer from unpredictable flare-ups and disease remission [8]. SLE diagnosis is currently based upon clinical and laboratory tests, and those results generate a Systemic Lupus Erythematosus Disease Activity Index (SLEDAI-2K) used to grade disease activity [8,64]. The pathophysiology of the disease involves increased activation of immune cells, overexpression of inflammatory cytokines, and autoantibodies along with immune complex deposition often in the kidney (lupus nephritis) [65]. There are several autoantibodies identified in SLE including anti-nuclear antibodies (ANAs), anti-extractable nuclear antigen (ENA), double-stranded DNA (dsDNA) antibodies, anti-SM, and anti-Ro; however, not all of them are found in all patients [37]. One of the criteria in SLEDAI-2K is the measurement of serum IgG against double-stranded DNA (anti-dsDNA), the most common antibody in the disease [65].

Dysfunction of B cells is common in SLE patients; more specifically, anti-dsDNA autoantibodies are produced both by long-lived plasmablasts in the bone marrow and short-lived ASCs derived from B_mem_ cells. Several studies have taken advantage of the role of B cells in SLE by targeting them as a potential treatment route. Rituximab, the anti-CD20 monoclonal antibody, is a successful treatment used in SLE to target B cells [66]. Even though anti-dsDNA autoantibody titers are measured in the SLE disease activity index (SLEDAI-2K) score to demonstrate disease, it is useful for some but not all patients [8,67,68].

There is a need for a more effective biomarker to track disease progression and predict flare-ups and the effectiveness of B cell depletion therapies [8]. The ELISPOT assay could be the solution, as it can enable tracking of spontaneous ASCs or B_mem_ cells with reactivity against autoantigens commonly targeted in SLE patients [67]. Hence, an ELISPOT assay could be devised with some of the following antigens: platelet membrane glycoprotein IIb-IIIa (gpIIb-IIIa), dsDNA, glutamic acid decarboxylase, and insulinoma antigen 2 [65]. A recent study by Perez-Isidro et al. reported the efficiency of an anti-dsDNA B cell ELISPOT assay as a biomarker of disease activity and suggested the assay as an additional biomarker to predict flare-ups in patients [65]. ELISPOT has also been used to measure CD27^+^IgD^+^ B cells, the main B-1 and innate-like B cells, and IgM producers, using the PBMC from 50 SLE patient samples, revealing a decreased IgM-producing ability of CD27^+^IgD^+^ B cells [63].

### 3.3. Graves’ Disease (GD)

Graves’ Disease (GD) is a multifactorial organ-specific autoimmune disorder that targets specifically the thyroid gland [69]. Although standard treatments such as antithyroid drugs (ATDs) can induce a temporary remission, the possibility of relapse is quite high, necessitating either radioactive iodine (RAI) or thyroid surgery to control the hyperthyroidism [70]. Disease diagnosis is confirmed by thyrotoxicosis (excess thyroid hormones), thyroid-stimulating hormone receptor (TSHR)-Ab positivity, goiter, and any associated pathology of eyes (orbitopathy) [71,72].

GD provides the ideal situation to test the utility of B cell ELISPOT in assessing the frequency of autoreactive B cells against TSHR. Thus far, several studies have utilized B cell ELISPOT assays in the context of GD but only for measuring overall (pan-specific) IgG, IgM, and IgA ASC activity [73,74,75]. Given that the disease is an archetypal example of an AD in which the effector antibodies are known, it provides the ideal disease for a B cell ELISPOT assay. B cell ELISPOT in the case of GD would not only provide valuable insight into the prognosis of the disease but also would enable monitoring the frequency of autoreactive B cells in the context of treatment [74,76].

It is well established that GD is a B cell-mediated AD that requires a breach in tolerance, which enables autoreactive B cells to produce antibodies against the thyroid [37]. A study by Y. Cao identified a subpopulation of B cells, CD11c^+^ B cells, that are increased in Graves’ Disease that exhibit an increased proinflammatory cytokine secretion profile (L-1β, IL-6, IL-17A, IFN-γ, and IL-9) compared to CD11c^-^ B cells [77]. These CD11c^+^ B cells expressed high levels of CXCR3 on their surface and are poised to become autoreactive B cells that produce TRAbs [78]. Given the role of TRAbs in the pathogenesis of GD, several studies have tried to target autoreactive B cells through B cell depletion therapies such as with Rituximab [38]. The use of Rituximab as a Graves’ Disease first line of treatment is not yet supported, as there are no major benefits compared to traditional treatment and cost of therapy is higher [38,71,79]. Even though Rituximab is a well-tolerated treatment for Graves’ orbitopathy patients, it does not decrease TRAb production or have an immediate effect on hyperthyroidism [38,79].

### 3.4. Hashimoto’s Thyroiditis (HT)

Hashimoto’s Thyroiditis (HT), also known as chronic lymphocytic or autoimmune thyroiditis, is an organ-specific AD and is the most prevalent thyroid AD [80]. Like Graves’ Disease it is heterogenous and can be caused by a combination of environmental factors and genetic susceptibility [81]. Even though both HT and GD target the thyroid, patients develop hypothyroidism and hyperthyroidism, respectively [69]. HT is driven by over-reactive T and B cells that target thyroglobulin and membrane-bound thyroid peroxidase on the thyrocytes [37,77]. As a result of the damaged follicular cells, there is insufficient production and secretion of thyroxine and triiodothyronine, the main thyroid hormones. Diagnosis of the disease uses clinical tests measuring serum antibodies against the thyroid and typical patchy hypoechoic appearance on ultrasound scans. Symptoms of the disease reflect the systemic effects of thyroid hormone deficiency including fatigue, somnolence, decreased cognitive function, myalgia, and fluid retention [81,82]. Patients that suffer from HT are usually treated with thyroxine hormone replacement for their hypothyroidism and receive symptom management.

B cells play a crucial role in the development and management of HT. The two types of antibodies that arise from the overactive B cells are thyroid peroxidase antibodies (TPOAbs) and thyroglobulin antibodies (TgAbs). A study by Nishihara et al. [83] compared the prevalence of TgAbs with TPOAbs for 70 patients using five different immunoassays and revealed that TgAbs were significantly increased compared to TPOAbs in HT patients. IgG4-related disease is common among HT disease patients from East Asia and is a fibroinflammatory disorder that involves increased swelling of the organs and increased production of IgG4 autoantibodies [84]. Clinicians have divided Hashimoto’s patients into either IgG4-positive or -negative based on the presence of lymphoplasmacytic cells in the tissue using an immunohistochemistry test [85]. Moreover, IgG4-positive HT patients are more likely to develop a subclinical manifestation of the disease compared to patients that are IgG4-negative [86]. The prospect of B cell depletion to selectively reduce diseases such as HT is becoming more popular. In a study by N. Ralchev et al., thyroglobulin-reactive B cells were suppressed with a chimeric protein molecule that provided inhibitory signals through the complement receptor CR1. Notably, the ELISPOT technique was used in this study to monitor the efficiency of the Tg1/2 chimeric molecule treatment in vitro [75].

### 3.5. Pemphigus Vulgaris (PV)

Pemphigus Vulgaris (PV) is a rare blistering disease of the mucous membranes and skin. The disease manifests when autoreactive B cells produce autoantibodies against desmoglein (DSG) 3 and 1 [87]. DSG 3 and 1 are calcium-dependent adhesion molecules that are part of the desmosome and are involved in maintaining tissue integrity. Autoantibodies against the desmosome are mainly of the IgG class and target cell-to-cell adhesions within the epidermis. The disruption in the structure triggers the formation of erosive lesions in the mucosa and the characteristic blister formation of the disease in the suprabasal layer of the skin seen in patients [44]. Furthermore, a hypothesis based on autoantibody deposition and distribution of desmoglein proteins exists known as Desmoglein Compensation Hypothesis, which proposes distinct phenotypes [88].

Autoantibody production in PV is a main diagnostic feature of the disease. The gold standard in diagnosing PV currently is direct or indirect immunofluorescence on tissue to detect IgG autoantibodies. Recently, there has been a compelling argument that C4b immunohistochemistry could also aid in diagnosis of PV in the esophagus as C4b (complement cascade product) is triggered by IgG and IgM antibodies [89].

Given the significant role of B cells in driving the disease, it is no surprise that B cell depletion therapies have been successful in treating the disease and establishing remission [90]. Rituximab, a B cell depletion therapy with the anti-human CD20 mAb, reduces the production of the autoantibodies and is now suggested as a first line therapy for PV. Undoubtably, by monitoring and quantifying autoreactive B cell frequency, there could be invaluable insights into PV disease development and treatment success. Nishifuji et al. detected autoreactive B cells directly ex vivo against DSG3 in PBMC. The results of the study revealed specific autoreactive B cells in the majority of PV patients but not in healthy controls [91]. Further studies could utilize the B cell ELISPOT assay in monitoring the patients while receiving treatment or during remission.

### 3.6. Anti-Phospholipid Antibody Syndrome (APS)

Anti-Phospholipid Antibody Syndrome (APS) is a systemic autoimmune disease that is three times more frequent in women compared to males. APS is characterized by the presence of anti-phospholipid antibodies (aPLs) that lead to arterial and venous thrombosis in addition to pregnancy complications in some cases [92]. There is a clear link between SLE and APS, and the risk of atherosclerosis and cardiovascular diseases is significantly higher in cases of patients that have these autoimmune disorders [93].

The role of B cells in APS development is central. Several circulating aPLs drive APS, which include both IgM and IgG antibodies against lupus anticoagulant (LAC), cardiolipin (CL), and β2-glycoprotein I (β2GPI) [94]. The autoantibodies in APS are targeted against phospholipids and the proteins that compose the lipid bilayer, which leads to the disruption of cell membranes. It is important to also note that memory B cells producing the same antibodies found in APS have also been identified in the blood of healthy non-autoimmune individuals [95]. B cell depletion therapies are not a first line therapy for APS. However, a study by Ji-Young Choi et al. has proven Rituximab to be successful in treating a case of APS nephropathy, paving the way for other drugs such as Eculizumab to also be tested [96].

B cell ELISPOT has yet to be established for any of the proteins that are targeted in APS (lupus anticoagulant, cardiolipin, and β2-glycoprotein I). Currently, there have been many studies focusing on the phenotype of the B cells involved in APS but not their specific functionality [50,95]. The establishment of such an assay could aid in the diagnosis of APS but also the assessment of the memory population of B cells that can be triggered to produce antibodies.

### 3.7. Autoimmune Pulmonary Alveolar Proteinosis (APAP)

APAP is a rare disorder that arises as a result of autoantibodies against granulocyte-macrophage colony-stimulating factor (GM-CSF) in the serum and lungs. GM-CSF autoantibodies (GMabs) block the function of GM-CSF and so inhibit the removal of pulmonary surfactant by alveolar macrophages, and hence the surfactant accumulates. Patients suffering from PAP accumulate surfactant in their alveoli and terminal bronchioles, causing dyspnea (difficulty breathing). GMabs exist at very low levels in the serum of healthy individuals to modulate multiple myeloid function. In APAP, IgG is the predominant isotype responsible for over 90% of antibodies compared to IgM and IgA. The results of a study by Nei et al. testing PBMC from APAP patients compared to healthy participants showed that B memory ASCs are responsible for IgG-GMAb. In this study, two methods were used to evaluate the PBMC samples, an ELISA and an ELISPOT assay, highlighting the difference between the two assays in the characterization of disease at a cellular level [97].

### 3.8. Myasthenia Gravis

Myasthenia Gravis is an AD that is antibody-driven and antibody-dependent. The antibodies associated with MG pathophysiology are antibodies generated against the nicotinic acetylcholine receptors (AChRs) at neuromuscular junctions. A minority of MG patients also have autoantibodies against MuSK and LRP4 or agrin. These antibodies activate complement and cause local damage through the depletion of acetylcholine, leading to muscle weakness and other morbidities. The different types of autoantibodies found in MG patients either to MuSK or AChR are critical to diagnosing MG patients to subtypes of the disease. For example, IgG1 and IgG3 autoantibodies are found in AChR MG patients, whereas IgG4 is the main antibody type in MuSK sero-positive MG patients [98]. Besides the effector autoantibodies mentioned above, striational autoantibodies against titin, ryanodine receptor, and the alpha subunit of the voltage-gated K+ channel can be measured as markers of disease severity [99].

There are several approved treatments for refractory generalized MG patients including intravenous immunoglobulins, plasma exchange, and immunoadsorption. Eculizumab, a humanized monoclonal antibody against complement protein C5 has also been approved for refractory generalized MG and has shown positive outcomes in patients [100]. Rituximab has shown efficacy in treating MG patients especially when they are MuSK antibody-positive. Some of the main benefits of using Rituximab was that it was fast-acting and well tolerated among patients. Recently, a drug known as Rozanolixumab, a high-affinity humanized immunoglobulin G4 monoclonal antibody that targets the human neonatal Fc receptor, was approved for the treatment of generalized MG as well. By targeting the human neonatal Fc receptor, Rozanolixumab decreases the levels of IgG including the autoantibodies detected in MG [31]. B cell ELISPOT has been optimized for identifying autoreactive B cells against AChR in mice using mononuclear cells from their draining lymph nodes and spleens in many reports; however, the assay has yet to be utilized effectively in the human setting [101]

## 4. Examples of Diseases in Which Autoantibodies Are Markers of Disease Pathogenesis

### 4.1. Multiple Sclerosis (MS)

Multiple Sclerosis (MS) is an autoimmune disease that affects the Central Nervous System (CNS), particularly the brain and spinal cord. Lesions develop in the areas of the brain due to increased demyelinating events and inflammation, which can be detected by an MRI test. The progression of the disease can be erratic and fluctuates from patient to patient. Early stages of the disease are described as an inflammatory state in which reversible neurological deficits are seen in patients, which eventually become permanent as white matter plaques accumulate in the CNS. Currently, the etiology of MS remains unclear and so diagnosis depends upon a process of eliminating other CNS conditions that can prove to be quite difficult in practice. The revised McDonald criteria from 2017 are used to confirm MS diagnosis using a combination of laboratory tests, imaging (MRI), and a physical examination [102]. Then, upon diagnosis, the patients are classed into four well-described types of MS: clinically isolated syndrome, relapsing–remitting MS, secondary progressive MS, and primary progressive MS [102,103].

Historically, MS has long been considered a T cell-mediated AD, specifically mediated by myelin-specific CD4^+^ T cells [19,104]. Newer insights into MS pathology has shown increased frequency of neuroantigen-reactive B memory present in MS patients [19], B cells to be a beneficial treatment route, and B cell depletion with anti-CD20 antibodies to be effective in treating the disease [3]. One of the most important obstacles clinicians face in treating MS patients is that currently there is no biomarker-predicting treatment outcome. Therefore, they base treatment on the four different demyelinating lesion patterns seen in MS patients. Recently, a large multicenter study by Levraut et al. sought to answer if blood and CSF-free light chains (kappa and lambda) proved to be reliable biomarkers in MS diagnosis when compared to healthy controls and patients with other CNS autoimmune disorders. The outcome of the study was that kappa CSF-free light chains (KFLCs) are a reliable marker of B cell activity in MS [105].

Kuerten et al. and Hohmann et al. were successful in identifying CNS-reactive B cells utilizing the B cell ELISPOT assay both directly ex vivo and after polyclonal stimulation using human brain lysate as antigen coated directly to the membrane [19,106]. A study by Rovituso et al. in 2015 [107], utilizing ELISPOT, was able to track brain-reactive B cells in the peripheral blood of MS patients. RRMS patients treated with glatiramer acetate responded to treatment better when they had increased numbers of brain-reactive B cells compared to patients who were treated with IFN-β [107]. A study by S. Kuerten et al. in 2020 [108] used the B cell ELISPOT assay to investigate its potential as a biomarker for MS. ELISPOT plates were coated with CNS antigen (tissue lysate), and MS patients’ PBMCs were stimulated with IL-2 and Toll-Like Receptor (TLR) 7/8 ligands, and after 5 days, the B cells were added to the plates to measure ASCs [108]. Similarly, a study by S. Tacke et al. successfully utilized B cell ELISPOT of cultured PBMCs to predict responses in MS patients that have relapsing–remitting MS undergoing glatiramer acetate or interferon β treatment. This study supports the utility of the assay in detecting reactive B cells against human brain lysate as a biomarker in MS [109].

### 4.2. Type 1 Diabetes (T1D)

Type 1 Diabetes (T1D) is a chronic autoimmune disease that is organ-specific to the pancreas, specifically involving the destruction of the insulin-producing Beta cells. T1D is a highly heterogenic disease that can be caused by combinations of genetic and environmental factors resulting in immunity (T cells and antibodies) against islet antigens. T1D is believed to be predominantly T cell driven. The specific antibodies that are present in the serum of the patients may be directed against insulin, glutamic acid decarboxylase (GAD), islet antigen-2 (IA-2), or the zinc transporter (ZnT8). Initial diagnosis is based on high serum antibody titers against any of the islet antigens, but these do not provide an accurate representation of ASC numbers or ongoing autoimmunity. B cell depletion with Rituximab is one of the suggested routes of therapy to downregulate the loss in insulin production by the islets in the patient’s pancreas. A study by Powell et al., in 2019 [67], investigated the role of B memory cells that recognize autoantigens and produce autoantibodies (GAD or IA-2 IgG antibodies) in patients that suffer from T1D. The ELISPOT assay confirmed a higher number of memory B cells in patients that are newly diagnosed with T1D compared to healthy individuals.

### 4.3. Primary Biliary Cholangitis (PBC)

In PBC, ELISPOT has been used to measure B cells secreting antibodies against the E2 component of the pyruvate dehydrogenase complex (PDC-E2) of mitochondria. These antibodies can inhibit enzyme activity and can cross-react with homologous environmental chemicals causing cirrhotic inflammation. The specific memory B cells and plasmablasts were found to be secreting predominantly IgM and IgA [110].

### 4.4. Acquired Hemophilia

Factor VIII of the clotting system can be targeted by antibodies in acquired hemophilia, which may inhibit the Factor VIII activity and worsen a patient’s clotting capability. This occurs mainly in young hemophiliacs receiving Factor VIII to treat their hemophilia. In addition, anti-factor VIII antibodies can occur in the elderly due to a breakdown in tolerance. In both groups, a better understanding of the dynamics of B cells making, or capable of making, these antibodies would assist clinical management of the disease. ELISPOT has been used to show anti-factor VIII antibody-secreting B cells in one study [111].

Table 2 indicates in which diseases ELISPOT has been used to measure memory B cells.

## 5. Limitations of the Assay

While the B cell ELISPOT assay is a valuable quantitative tool for ASC measurement, several limitations must be considered. ELISPOT also requires testing (and potentially cryopreservation and recovery) of viable cells, compared to serum-/plasma-based measurements, which have less stringent sample collection/maintenance requirements [16]. In order to study memory B cells using the ELISPOT technique, such cells are required to transition into antibody-secreting cells (ASCs) to enable their detection based on the resulting antibody-derived secretory footprint. The B cell ELISPOT assay has shown great promise as a diagnostic tool in detecting ASCs against a given Ag when an individual has been recently vaccinated, provided the cells are spontaneously secreting antibodies due to vaccination or infection [108,112]. Another limitation of ELISPOT is the need to perform prior stimulation to transition the cells into ASCs to facilitate their detection.

In the realm of autoimmunity, such antigen-specific memory B cells would ideally be detectable in PBMC. However, it remains plausible that such autoreactive, antigen-experienced B cells may primarily reside in the tissue (e.g., the thyroid in GD or the pancreas for T1D) during the earliest phases of disease initiation, when medical intervention via depletion of B cells would be most effective [76,113]. Furthermore, the assay also requires the identification of antigen(s) of interest and then achieving adequate coating (if the coating is suboptimal, the SFU on the membrane may not be easily distinguishable) [16]. Moreover, in most B cell-mediated ADs, the target autoantigens have been identified, but few of them are presently commercially available as recombinant proteins. This is presently limiting progress in this field, as such antigens are required for rapid progress of ELISPOT as a B cell immunodiagnostic [20]. While the assay can quantify the Ag-specific ASCs, it does not allow for physical isolation of the cells for sequencing or transcriptomics. However, if they are detected in ELISPOT, we know that these cells are present in the sample and could be subjected to more detailed characterization such as phenotyping the B cell compartment by antigen probe-based flow cytometry. Focusing on a particular antigen or set of antigens for assessment of antigen-specific B cell reactivity is also imperative. Whereas serum is rarely a limiting reagent for serological assays, and sufficient serum is available for a battery of antibody tests, cell material requirements and specifically the number of viable PBMCs required to detect antigen-specific memory B cells, especially when they exist at very low frequencies, could be a limitation.

## 6. Advantages of the Assay over Existing Tools

The B cell ELISPOT assay is easy to perform and provides single-cell resolution of individual autoreactive ASCs, assuming antigen coating is adequate [16]. ELISPOT also allows for detection of antigen-specific B cells that have not terminally differentiated in vivo and hence have not contributed to serum/plasma antibody reactivity. B cell effector function in autoimmunity is not always via secretion of antibody, as in T1D where B cells play a critical APC function. Since T/B cell interactions are bi-directional, evidence of clonally expanded and class-switched autoreactive B cells (especially in the memory B cell compartment) may also be a more sensitive indicator of T cell-derived autoimmune conditions. In addition to the increased diagnostic specificity, detection of autoantigen-specific plasmablasts by ELISPOT may permit the early detection of flares of the disease. Spontaneously-secreting plasmablasts occur in blood only during phases of actively ongoing immune responses, when B cells primed in lymphoid tissues migrate to niches where they become sessile, e.g., the bone marrow. Such plasmablasts can be detected in direct ELISPOT assays, in which the PBMCs are plated without additional pre-activation. In Kuerten et al. [19], indeed, such plasmablasts were detected in patients during exacerbation of the disease. Identifying periods of immune activity should be critical to better treatment of B cell-mediated autoimmune diseases, as these are the time points during which intensive immune suppressive therapy is indicated, and for most autoimmune diseases such exacerbations are short compared to the long inactive periods in between these episodes.

The detection of B memory cells in general seems to offer higher diagnostic specificity, even with foreign antigens, such as those of preceding viral infections; the detection of memory B cells being a more reliable indicator of a primed state in some instances than the detection of serum antibodies [14]. This is because serum antibodies are short-lived molecules that are diluted into all bodily fluids, whereas memory B cells are long-lived and recirculate in the blood steam. The detection of autoantibodies is further complicated by the fact that their concentration in serum further decreases as they are absorbed by the autoantigen. Evidence for this was seen for Multiple Sclerosis, where neuroantigen-specific memory B cells could be readily detected by ELISPOT, yet not serum antibodies [108]. A more sensitive method for detecting the induction of autoreactive B cells may help with diagnosing B cell-mediated autoimmune diseases in their earliest phases, as was seen with clinically isolated syndrome (CIS) patients, the earliest form of MS [19]. Such early diagnosis, in turn, should enable treatment before irreversible damage is caused by the autoimmune process.

Recent advances are making the B cell ELISPOT assay universally applicable for any recombinant antigen [20], and the robustness of the assay [16] plus the need for cellular immune monitoring versus solely serum diagnostics are enhancing the prospects of its uptake as an immunodiagnostic [16]. These advances are as follows: (1) Affinity capture coating using “tagged” antigens to achieve higher-density coating (necessary for detection of ASCs with modest affinities for the antigen); (2) inverted assays that leverage the same “tagged” antigens to delve into specific Ig class or IgG subclasses (if known to associate with detrimental disease progression/patient outcome) or delve further into affinity maturation (probe titration); (3) objective, user-independent image analysis algorithms for assay quantification [114]; and (4) high-content data analysis tools provided by ImmunoSpot^TM^software to “go beyond just counting spots”. These are all properties underpinning developments in ELISPOT.

## 7. Additional Applications

The enzyme-linked detection assays (ELISPOT) can be readily multiplexed using fluorescence-based detection (FluoroSpot) of B cell secretory footprints. Both assays have similar sensitivity [16,115], but multicolor assays permit the simultaneous detection of different Ig classes and subclasses within the antigen-specific B cell repertoire, and as these mediate different downstream effector functions (like complement activation and opsonization), their characterization provides closer insights about the expected biological consequences that binding of autoantibodies will exert. As multiplexed assays utilize the same amount of cells as single-color assays, the former are saving cell material needed for testing.

Owing to limitation in cellular material, as well as the potential for large differences in the frequency of ASCs producing various Ig classes or IgG subclasses, the fluorescent version of the assay (FluoroSpot) could offer additional resolution for dissecting the B cell/ASC response [116]. Modified ELISPOT, such as the inverted assay, could also be useful in at least two scenarios. First, using an inverted assay approach, multiple autoantigens could be pooled simultaneously, especially for screening purposes in which precise quantification of ASC reactivity against individual autoantigens is not a priority. Second, the inverted approach (“modified” assay; Figure 2) could also provide information on the functional affinity of specific autoreactive B cell populations through simple probe titration, and may provide further resolution into the autoimmune disease course/progression beyond only measuring whether the frequency of such autoreactive cells increased. The primary goal of this review was to advocate for the use of B cell ELISPOT into autoimmune diagnostics and to provide a survey of the existing, but limited, literature. “OMICS” technologies, especially those providing single-cell resolution, have certainly shed critical insights into the transcriptional signature of B cells (and other cell types) in the context of autoimmunity. There are many ways in which ELISPOT and OMICS technologies could be combined to provide deeper insights into the initiation or severity of autoimmune diseases. For example, ELISPOT could be useful for identifying patient samples (PBMC or single-cell suspensions from the affected tissue/organ) that possess autoreactive ASCs—either spontaneously or following polyclonal stimulation—that could be then subjected to single-cell RNA sequencing. Such work, especially in the context of disease initiation or treatment, may reveal insightful positive or negative associations.

Alternatively, and coming from the other side, the inverted ImmunoSpot assay approach could be used to evaluate the incidence of autoreactive ASCs (spontaneous or following polyclonal stimulation), targeting a specific autoantigen identified by mass spectrometry (after IgG pull down, for example) in larger patient cohorts. Such an approach would be far more efficient for confirming such observations/associations and would further allow for direct enumeration of such ASCs and evaluation of their Ig class/IgG subclass usage or affinity.

## 8. Conclusions

Currently, a major challenge in autoimmune disease management is the lack of biomarkers that are able to accurately track disease progression and response to treatments (experimental [drug trials] and routine). Typically, ELISAs are used in most ADs to measure antibodies in the patient’s serum, which are considered to be biomarkers of an AD. It is important to distinguish between diseases in which autoantibodies are known to be prime mediators of the disease, those in which they participate in disease (where B cell depletion succeeds), and where they are just associated with the disease, i.e., surrogate biomarkers [101]. Ultimately, ELISAs are restricted to measuring the serum antibodies produced in vivo by the specific long-lived plasma cells, whereas B cell ELISPOT can measure the B_mem_ cells upon polyclonal stimulation. The latter is likely to reveal the more durable component of the immune response that requires monitoring and treating and is targeted by B cell-depleting therapies such as anti-CD20 monoclonal antibodies. Undoubtedly, the biggest challenge will be identifying the most appropriate autoantigen to be used in the assay, especially in ADs in which the pathophysiology of the disease depends upon immunity against multiple autoantigens. Despite the fact that more than a hundred autoantibodies have been identified of different specificities for ADs, screening assays are often not sensitive enough to identify autoantigens to which autoantibodies are created in the AD. B cell ELISPOT may provide a valuable addition or alternative to serum antibody measurement.

## Figures and Tables

**Table 1:** The main autoimmune diseases in which anti-self antibodies are considered important as disease-causing effectors or markers of disease.

Target Tissue/ Organ	Autoimmune Disease	Examples of Main Target Antigens/Antibodies (Not Exhaustive)	Effector or Marker Antibodies?	B Cell-Targeted Therapy Given?
Bones and Joints	Rheumatoid Arthritis	Rheumatoid factor (anti-IgGFc), Anti-MCV, ACPAs, Vimentin	Effector and marker [27]	Rituximab phase III [2]
Nervous System	Multiple Sclerosis	Potassium channel, MOG, Anoctamin-2, Myelin Basic Protein, Anti-Kir4.1, Anti-ANO2	Marker	Rituximab Ocrelizumab Ofatumumab [1]
Nervous System	Myasthenia Gravis	Nicotinic acetylcholine receptor, Muscle-specific kinase (MuSK), LRP4 (low-density lipoprotein receptor-related protein 4), Agrin [28]	Effector and marker	Eculizumab [29] Rituximab [30] Rozanolixizumab [31]
Nervous System	Other neuropathies: Guillain–Barré Syndrome, Miller-Fisher Syndrome	Ganglioside antibodies: anti-GM1, GD1a, GQ1b; Alpha-enolase, GQ1b; Yo (cdr-2 in Purkinje fibers) Hu, Tr, glutamate receptor	Effector and marker [32]	Eculizumab phase II trial [33]
Nervous System	Ocular Neuromyelitis Optica Spectrum Disorder (NMOSD)	Aquaporin 4	Marker	Rituximab [34]
Nervous System	Stiff person syndrome	GAD-65	Effector	Rituximab was ineffective [35]
Endocrine	Graves’ Disease	Thyroid autoantibodies (TSHR-Ab) that activate the TSH receptor (TSHR) IGF-1 receptor [36,37]	Effector and marker	Rituximab [38]
Endocrine	Hashimoto’s Thyroiditis	Thyroid peroxidase and/or thyroglobulin	Marker	
Endocrine	Type 1 Diabetes	Glutamic acid decarboxylase (GAD), islet cell antigen (ICA), insulinoma-associated (IA-2), insulin, ZnT8 [39]	Marker	Rituximab was unsuccessful [9]
Endocrine	Addison’s Disease (Adrenal)	21 hydroxylase	Marker	Rituximab [40]
Endocrine	Autoimmune pancreatitis	ANA, lactoferrin S, anti-carbonic anhydrase, rheumatoid factor	Marker	Rituximab [41]
Kidney	Nephritis	Basement Membrane Collagen Type IV protein	Marker and effector	Combination therapy with Rituximab [42]
Kidney	IgA nephropathy, Henoch–Schönlein purpura	IgA1-Glycan	no	no
Skin	Pemphigoid	Type XVII collagen component of hemidesmosomes; BP-1, BP-2	Marker and effector	Rituximab [43]
Skin	Pemphigus Vulgaris	Desmoglein 3 Desmoglein 1	Effector [44]	Rituximab [45]
Skin	Scleroderma	Anti-nuclear antibodies, centromere and scl70/anti-topoisomerase	Marker	Belimumab Rituximab [24,25]
Liver	Primary Sclerosing Cholangitis	ANA, smooth muscle, ANCA	Marker	Rituximab phase II [46]
Liver	Primary Biliary Cholangitis	p62, sp100, Mitochondrial (M2), Ro AKA SSA PDC-E2	Marker	Rituximab is investigated for fatigue: unsuccessful [47]
Liver	Autoimmune hepatitis	ANA and SMA, LKM-1, LKM-2, or LKM-3; soluble liver antigen	Marker	Rituximab third line treatment
Multi-Systemic	Sjögren’s Syndrome	Ro/SS-A and La/SS-B	Effector and marker	Rituximab [5]
Multi-Systemic	Systemic Lupus Erythematosus	Anti-nuclear antibodies (ANAs), anti-extractable nuclear antigen (ENA), double-stranded DNA (dsDNA) antibodies, anti-SM, anti-Ro [48]	Effector and marker	Rituximab Belimumab Epratuzumab [8]
Multi-Systemic	Granulomatosis, Polyangiitis, Vasculitis	cANCA, pANCA, C1q, IgA, and complement component 3	Effector and marker	Rituximab [49]
Multi-Systemic	Anti-Phospholipid Antibody Syndrome (APS)	Cardiolipin, β2-glycoprotein I, and Lupus anticoagulant [50]; HPA-1a, HPA-5b	Effector and marker [50]	no
Multi-Systemic	Thrombocytopenia	GpIIb-IIIa or 1b-IX; glycoproteins IIb-IIIa or Ib-IX in ITP ADAMTS13 in TTP; and HUS	Effector and marker	Rituximab second line treatment [51]
Lung	Anti-synthetase syndrome	Aminoacyl tRNA Synthetase: Jo1, PL7, PL12	Marker	Rituximab [52]
Lung	Autoimmune Pulmonary Alveolar Proteinosis	GM-CSF	Effector and marker	no
Gut	Celiac Disease	Tissue transglutaminase antibodies, endomysial IgA, gliadin IgA	Effector and marker [53]	no
Heart	Myocarditis, Eosinophilic granulomatosis with polyangiitis (EGPA)	Myocardial antigens, cardiac myosin s, and β1-adrenergic receptor	Marker [54]	Rituximab [55]

(See text or reference for abbreviations.)

**Table 2:** Diseases in which B cell ELISPOT has been recently used to detect autoantigen-specific human B cells.

Autoimmune Disease	Ex Vivo or Pre-Cultured ELISPOT
Multiple Sclerosis	(1) Pre-cultured ELISPOT (antigen directly coated onto plate) [109] (2) Pre-cultured ELISPOT to detect B_mem_ cells when the plates are coated directly with whole human lysate [19]
Rheumatoid Arthritis	(1) Pre-cultured ELISPOT (β60–74 peptides represent the major ACPA epitope on the β chain of fibrin antigen directly coated onto plate) [59] (2) Pre-culture for 14 days ELISPOT (biotinylated antibody, goat (Fab2)–anti-IgM (10 μg/mL)) to measure IgM–anti-CCP-secreting ASCs in bone marrow and synovial fluid [57] (3) Directly ex vivo measuring plasmablasts plate-coated anti-IgG–biotin (MT78/145, Mabtech), biotinylated CCP2 peptide, or its arginine control variant (CArgP2) [62]
Hashimoto’s Thyroiditis	Plates pre-coated with 5 μg/mL Tg1/2 peptide antigen directly coated onto plate to measure plasmacytes, not B_mem_ [75]
Acquired Hemophilia	An inverted ELISA assay capturing all Igs and then probing with recombinant Factor VIII labeled with AF555
Graves’ Disease	Coated with goat anti-mouse IgM antibody to measure plasmablasts/no incubation [73]
Type 1 Diabetes	Pre-coated with islet antigens such as insulin, glutamic acid decarboxylase GAD- and IA-2, stimulating B_mem_ [67]
Pemphigus Vulgaris	Pre-coated with rDsg3-his B_mem_ stimulation [91]
Systemic Lupus Erythromatosus	(1) Directly ex vivo coated with 100 μL calf thymus DNA to measure anti-dsDNA [65] (2) Pre-cultured to measure IgM-producing CD27+IgD+ B cells in active SLE [63]

## Data Availability

No new data were created or analyzed in this study.

## Abbreviations

B_mem_	B memory cells
ELISPOT	Enzyme-linked ImmunoSpot assay
AD	autoimmune disease
ASC	antibody-secreting cell
B_mem_	B memory
BCR	B cell receptor
RA	Rheumatoid Arthritis
SS	Sjögren’s Syndrome
MG	Myasthenia Gravis
UC	Ulcerative Colitis
SLE	Systemic Lupus Erythematosis
PVDF	polyvinylidene fluoride
ELISA	enzyme-linked immunoassay
EBV	Epstein–Barr Virus
CMV	Cytomegalovirus
T1D	Type 1 Diabetes
BAFF	B cell-activating factor
ANAs	anti-nuclear antibodies
ENA	anti-extractable nuclear antigen
dsDNA	double-stranded DNA
GAD	glutamic acid decarboxylase
ICA	islet cell
IA	insulinoma-associated
APF	anti-perinuclear factor
AKA	anti-keratin
ESR	erythrocyte sedimentation rate
CRP	C-reactive protein
ATD	antithyroid drug
TSHR	thyroid-stimulating hormone receptor
TRAbs	anti-thyrotropin receptor antibodies
TSAbs	thyroid-stimulating antibodies
TBAbs	thyroid-blocking antibodies
TPOAbs	thyroid peroxidase antibodies
TgAbs	thyroglobulin antibodies
LAC	anticoagulant
CL	cardiolipin
β2GPI	β2-glycoprotein I
AchR	acetylcholine receptor
MuSK	muscle-specific kinase
LRP4	low-density lipoprotein receptor-related protein 4
GAD	glutamic acid decarboxylase
IA-2	islet antigen-2
ZnT8	the zinc transporter
CNS	Central Nervous System
TLR	Toll-Like Receptor
PBC	Primary Biliary Cholangitis
PDC-E2	pyruvate dehydrogenase complex
